# A SARS-CoV-2 vaccine candidate would likely match all currently circulating variants

**DOI:** 10.1073/pnas.2008281117

**Published:** 2020-08-31

**Authors:** Bethany Dearlove, Eric Lewitus, Hongjun Bai, Yifan Li, Daniel B. Reeves, M. Gordon Joyce, Paul T. Scott, Mihret F. Amare, Sandhya Vasan, Nelson L. Michael, Kayvon Modjarrad, Morgane Rolland

**Affiliations:** ^a^Emerging Infectious Diseases Branch, Walter Reed Army Institute of Research, Silver Spring, MD 20910;; ^b^US Military HIV Research Program, Walter Reed Army Institute of Research, Silver Spring, MD 20910;; ^c^Henry M. Jackson Foundation for the Advancement of Military Medicine, Bethesda, MD 20817;; ^d^Center for Infectious Diseases Research, Walter Reed Army Institute of Research, Silver Spring, MD 20910;; ^e^Vaccine and Infectious Disease Division, Fred Hutchinson Cancer Research Center, Seattle, WA 98109

**Keywords:** SARS-CoV-2, evolution, vaccine

## Abstract

The magnitude of the COVID-19 pandemic underscores the urgency for a safe and effective vaccine. Many vaccine candidates focus on the Spike protein, as it is targeted by neutralizing antibodies and plays a key role in viral entry. Here we investigate the diversity seen in severe acute respiratory syndrome coronavirus 2 (SARS-CoV-2) sequences and compare it to the sequence on which most vaccine candidates are based. Using 18,514 sequences, we perform phylogenetic, population genetics, and structural bioinformatics analyses. We find limited diversity across SARS-CoV-2 genomes: Only 11 sites show polymorphisms in >5% of sequences; yet two mutations, including the D614G mutation in Spike, have already become consensus. Because SARS-CoV-2 is being transmitted more rapidly than it evolves, the viral population is becoming more homogeneous, with a median of seven nucleotide substitutions between genomes. There is evidence of purifying selection but little evidence of diversifying selection, with substitution rates comparable across structural versus nonstructural genes. Finally, the Wuhan-Hu-1 reference sequence for the Spike protein, which is the basis for different vaccine candidates, matches optimized vaccine inserts, being identical to an ancestral sequence and one mutation away from the consensus. While the rapid spread of the D614G mutation warrants further study, our results indicate that drift and bottleneck events can explain the minimal diversity found among SARS-CoV-2 sequences. These findings suggest that a single vaccine candidate should be efficacious against currently circulating lineages.

## Significance

The rapid spread of the virus causing COVID-19, SARS-CoV-2, raises questions about the possibility of a universally effective vaccine. The virus can mutate in a given individual, and these variants can be propagated across populations and time. To understand this process, we analyze 18,514 SARS-CoV-2 sequences sampled since December 2019. We find that neutral evolution, rather than adaptive selection, can explain the rare mutations seen across SARS-CoV-2 genomes. In the immunogenic Spike protein, the D614G mutation has become consensus, yet there is no evidence of mutations affecting binding to the ACE2 receptor. Our results suggest that, to date, the limited diversity seen in SARS-CoV-2 should not preclude a single vaccine from providing global protection.

Severe acute respiratory syndrome coronavirus 2 (SARS-CoV-2), the virus that causes COVID-19, is a member of the Coronaviridae family, a diverse group of virus species, seven of which are known to infect humans. Four are considered endemic and typically cause mild upper respiratory illnesses; two of these, NL63 and 229E, are within the alphacoronavirus genus, and two, HKU1 and OC43, are betacoronaviruses. The latter genus comprises the three highly pathogenic human coronaviruses, including SARS-CoV-2, as well as Middle Eastern respiratory syndrome (MERS) CoV and severe acute respiratory syndrome (SARS) CoV. SARS-CoV is the most closely related human virus to SARS-CoV-2, which is a single-stranded positive-sense RNA virus, with an ∼30,000-base pair genome. The genome is split into 10 open reading frames (ORFs) that include 16 nonstructural proteins and four structural proteins. The latter category includes Spike (S), Membrane (M), Envelope (E), and Nucleocapsid (N). S is the basis for most candidate vaccines, as it mediates virus attachment and entry to host cells and is the target of neutralizing antibody responses (1–4). S is cleaved into two subunits, S1 and S2: The former contains the receptor binding domain (RBD), which enables the virus to attach to the angiotensin-converting enzyme 2 (ACE2) receptor on host cells.

In the span of 7 months, the COVID-19 pandemic has caused a devastating global health crisis with significant mortality and socioeconomic implications. As of July 23, 2020, more than 15 million cases and 622,000 attributable deaths have been reported worldwide (5–8) (https://coronavirus.jhu.edu/map.html). Phylogenetic analyses suggest that SARS-CoV-2 is likely derived from a clade of viruses found in horseshoe bats (9). In S, the bat genome RaTG13 has more than 97% amino acid identity with SARS-CoV-2 (6). Interestingly, the RmYN02 sequence, which is the closest to SARS-CoV-2 in the long ORF1ab but more distant than RaTG13 in S, showed the insertion of multiple amino acids at the cleavage site between the S1 and S2 subunits of the S protein (this S1/S2 insertion is a characteristic feature of SARS-CoV-2) (10). Highly similar sequences, especially in the RBD, were also identified in Malayan pangolins (11, 12), emphasizing the plasticity of coronavirus genomes and their propensity to switch hosts. Although the closest currently available bat sequences are fairly divergent from SARS-CoV-2, their characteristics (insertion at S1/S2 cleavage site, high diversity, and similarity between specific gene fragments and particular strains) together with their known adaptive properties (high recombination and host-switching rates and evidence of positive selection) support that these bat viruses constitute a generalist lineage where a specific virus is likely the natural origin of SARS-CoV-2. We did not study the transmission of the virus from its animal reservoir and focused our analysis on the evolution of SARS-CoV-2 since its introduction in humans. While the scale of the pandemic attests to the high transmissibility of SARS-CoV-2 between humans, with a basic reproduction number R_0_ estimated to be 2.2 (95% CI, 1.4 to 3.9) in Wuhan, China (13), we wanted to investigate evidence of further adaptation of SARS-CoV-2 to its host, as adaptive processes could interfere with vaccine efficacy.

Developing a vaccine against SARS-CoV-2 is a high priority for preventing and mitigating future waves of the pandemic (14). Vaccine candidates typically include an insert that corresponds to one or more virus antigens, either derived computationally or from one or multiple sequence(s) sampled from infected individuals. The first viral sequence derived during the COVID-19 outbreak, Wuhan-Hu-1 (available from the Global initiative on sharing all influenza data, GISAID, accession EPI_ISL_402125), was published on January 9, 2020. As many vaccine programs were initiated at that time, it is likely that this SARS-CoV-2 sequence, sampled in December 2019 in Wuhan, China, is the foundation for many vaccine candidates currently in development. Compared to other RNA viruses, coronaviruses have a more complex molecular machinery resulting in higher replication fidelity. Early evolutionary rate estimates for SARS-CoV-2 were ∼1 × 10^−3^ substitutions per nucleotide per year (15), a rate comparable to that observed during the SARS-CoV-1 outbreak (16) and in the range for other RNA viruses (1 × 10^−3^ to 1 × 10^−5^ substitutions per nucleotide per year) (17). While the evolutionary rate is likely to decrease over time (18), it is important to monitor the introduction of any mutation that may compromise the potential efficacy of vaccine candidates derived from the first available SARS-CoV-2 sequences.

New mutations will be observed as the virus spreads in humans. The viral evolutionary dynamics can be characterized by analyzing viral sequences sampled from individuals who became infected. The accumulation of mutations can be a marker of viral fitness: An increase in viral fitness as the virus adapts to its host will be associated with pervasive mutations at specific sites, whereas a neutral evolution context will be associated with a minimal number of fixed mutations distributed stochastically across the genome. Indicators of viral evolution have been shown to be robust predictors of transmission dynamics for several pathogens, such as influenza (19), Lassa (20), and Ebola (21) viruses. Typically, the evolution of a virus is driven by genotypic and phenotypic changes in its surface protein. In the case of SARS-CoV-2, mutations in S are most likely to confer fitness to the virus as it adapts to humans. However, adaptive changes can occur in structural and nonstructural proteins, and these changes, as well as different patterns across structural and nonstructural proteins, may provide insights into the near- and long-term evolutionary dynamics of SARS-CoV-2, as it spreads in humans. Here we analyzed SARS-CoV-2 sequences sampled since the beginning of the pandemic and found that mutations were rare, indicating that potential vaccine candidates should cover all circulating variants.

## Results

### Limited Diversity across 18,514 SARS-CoV-2 Genomes.

**Fig. 1.**
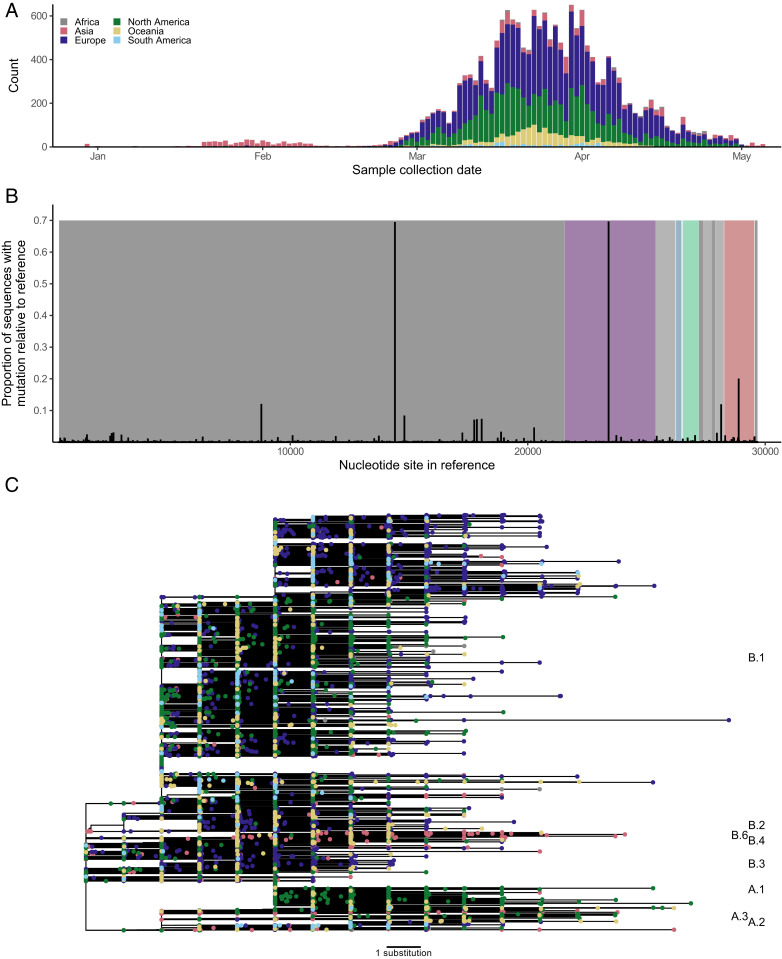
SARS-CoV-2 diversity across 18,514 genomes. (*A*) Distribution representing the location and date of sample collection. (*B*) Location and frequency of sites with polymorphisms across the genome. Proportion of sequences that showed polymorphisms compared to the reference sequence, Wuhan-Hu-1 (GISAID: EPI_ISL_402215, GenBank: NC_045512). ORFs are shown in gray for nonstructural proteins and in color for structural proteins (S, purple; E, blue; M, green; N, red). (*C*) Global phylogeny of 18,514 independent genome sequences. The tree was rooted at the reference sequence, Wuhan-Hu-1, and tips are colored by collection location. The scale indicates the distance corresponding to one substitution. Lineages are labeled following PANGOLIN (22).

To characterize SARS-CoV-2 diversification since the beginning of the epidemic, we aligned 27,977 SARS-CoV-2 genome sequences isolated from infected individuals in 84 countries. The alignment was curated to retain independent sequences that covered over 95% of the ORFs. In addition, because sequences from the United Kingdom constituted 47% of the dataset (*n* = 12,157), we sampled a representative set of 5,000 UK sequences, yielding a final dataset of 18,514 SARS-CoV-2 genomes (*SI Appendix*, Fig. S1 and Fig. 1*A*).

There were 7,559 polymorphic sites (that is, sites where at least one sequence has a change relative to the reference sequence) across the genome (total length: 29,409 nucleotides). Most substitutions were found in a single sequence; only 8.41% (*n* = 2,474) of the polymorphic sites showed substitutions in two or more sequences (Fig. 1*B*). Only 11 mutations were found in >5% of sequences, and only 7 were found in >10% of sequences (3 of which were adjacent). The mean pairwise diversity across genomes was 0.025%, ranging between 0.01% for E to 0.11% for N. A phylogenetic tree reconstructed based on all genome sequences reflected the global spread of the virus: Samples from the first 6 wk of the outbreak were collected predominantly from China (Fig. 1*C*). As the epidemic has progressed, samples have been increasingly obtained across Europe and from the United States (Fig. 1 *A* and *C*). The tree shows numerous introductions of different variants across the globe, with introductions from distant locations seeding local epidemics, where infections sometimes went unrecognized for several weeks and allowed wider spread (23). Prior to the severe travel restrictions that were seen in March 2020, intense travel patterns between China, Europe, and the United States allowed transmission of a myriad of variants, which is currently reflected by different lineages in the tree. Yet, the tree topology shows minimal structure, even at the genome level, indicating that SARS-CoV-2 viruses have not diverged significantly since the beginning of the pandemic. To compare how genomes differed from one another, we calculated Hamming distances (which correspond to the number of differences between two genomes) across all pairs of sequences. These Hamming distances showed a narrow distribution, with a median of seven substitutions between two independent genomes, while linked sequences sampled in cruise ships had a median of two substitutions (*SI Appendix*, Fig. S2). Surprisingly, Hamming distances across genomes sampled in the United States did not show a similar quasi-normal distribution but instead a bimodal distribution, observed despite the large number of sequences compared (*n* = 5,398). We identified that this bimodal distribution was driven by sequences from Washington State, possibly reflecting separate introductions in that state. Nonetheless, such a bimodal distribution could also indicate a bias in the sampling of sequences (*SI Appendix*, Fig. S2).

### One S Mutation (D614G) Has Become Dominant.

**Fig. 2.**
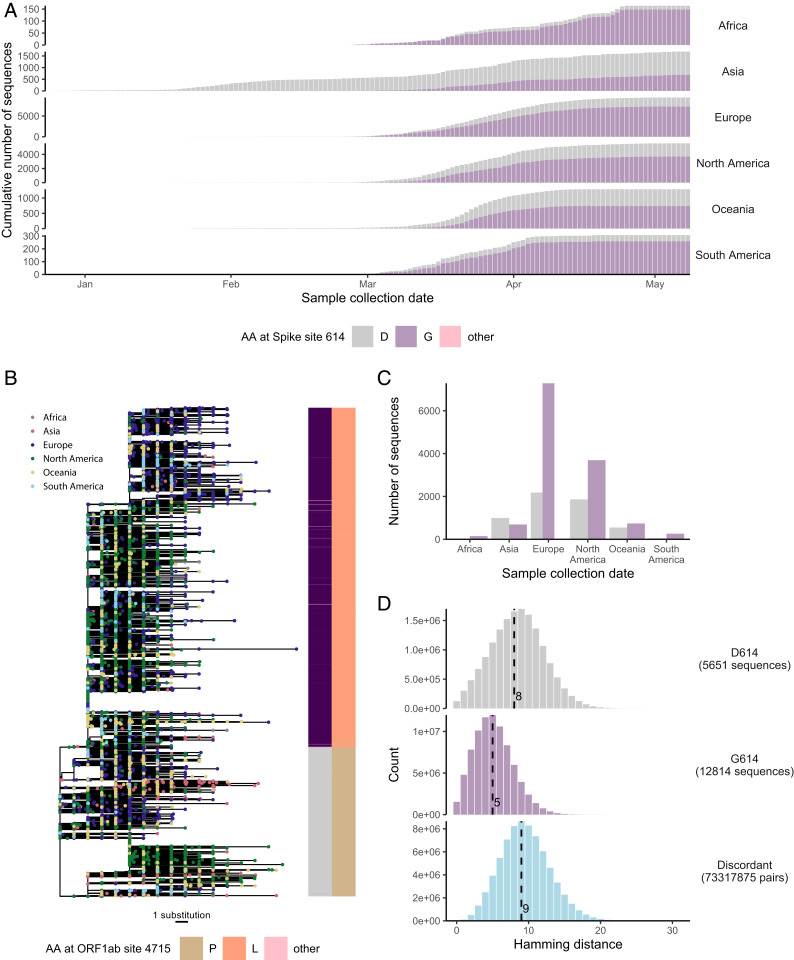
The S mutation D614G quickly became dominant. The mutation D614G was found in 69% of sequences sampled globally as of May 18, 2020, the second most frequent mutation in S was only found in ∼2% of sequences. (*A*) Number of sequences with D (gray) or G (purple) by continent and sampling date shown cumulatively through the outbreak. (*B*) Phylogenetic tree reconstructed from all of the ORFs showing the linkage between D614G in S and P4715L in ORF1ab. Tips are colored by continent. The phylogeny suggests that these mutations were linked to a bottleneck event when SARS-CoV-2 viruses were introduced in Europe; this mutation was first seen in Europe in a sequence sampled in Germany at the end of January. There is no evidence that the increasing predominance of this mutation was caused by convergent selection events that would have occurred in multiple individuals. (*C*) Overall number of sequences with D614 or D614G across continents; the predominance of D614G in Europe is suggestive of a founder event. (*D*) Distribution of Hamming distances between sequences with D614, G614 or discordant pairs. The median is marked with a dashed line.

Since the beginning of the pandemic, two mutations across the genome have become consensus: P4715L in ORF1ab (nucleotide 14,143, C to T) and D614G in S (nucleotide 23,403, A to G) (Fig. 1*B*) (a third consensus mutation, at nucleotide 3,037, is not reported as the site was masked during our sequence-filtering procedure). These mutations were found in 69.3% and 69.4% of sequences, respectively, and are in linkage (Fig. 2*B*). Given the importance of S for virus entry and as a target of the host neutralizing response, the biologic implications of the D614G mutation are under intense scrutiny (24–28). This mutation was first observed in a sequence from China dated January 24, with seven more sequences sampled until February 8. Then, the D614G mutation was not observed in China until March 13. In contrast, the D614G mutation was introduced in Europe at the end of January (first sequence identified in Germany, dated January 28), and it rapidly became dominant on that continent and at every location where the virus subsequently spread (Fig. 2*A*). The phylogenetic tree (Fig. 2*B*) and the distribution of sequences (Fig. 2*C*) are suggestive of a founder effect. The rapid spread of sequences carrying the D614G mutation implies that the growing viral population should become more homogeneous, that is, the frequency of sequences with shared polymorphisms will increase. We found a median of seven substitutions (based on a comparison of 18,514 sequences) between two independent SARS-CoV-2 genomes (*SI Appendix*, Fig. S2). Yet, genomes with the D614G mutation showed a median of five substitutions, whereas those with D at position 614 differed by eight substitutions (Fig. 2*D*).

To test whether this site was under selection, we used likelihood-based, phylogenetically informed models that assume branch-specific substitution rates (29) and implemented a sampling strategy to circumvent computational limitations imposed by the large number of sequences. Subsampled alignments (100 times at a 10% sampling fraction) had diversity estimates statistically similar to the complete alignment for each gene (Mann−Whitney *U* test, *P* > 0.09; *SI Appendix*, Fig. S3). In S, only site 614 was estimated to be under diversifying selection in a majority of subsampled alignments (58%); evidence of diversifying selection indicates that genetic diversity increases in the viral population (i.e., there was a higher proportion of mutations causing an amino acid change than not at site 614, or, the nonsynonymous/synonymous substitution rates ratio, dN/dS, was over 1, *P* < 0.1) (*SI Appendix*, Fig. S4). Because diversifying selection is often associated with the host adaptive response, we considered whether the D614G mutation coincided with targets of antibody and T cell responses. Site 614 is at the interface between the S1 and S2 subunits and is thus not highly accessible to antibodies (*SI Appendix*, Fig. S5). Therefore, we predict that antibodies to the native S protein would cross-react with S containing the D614G mutation, in agreement with recent reports (24, 25, 27, 28). Many known neutralizing antibodies target the RBD, yet we found little evidence that mutations could affect binding to the ACE2 receptor, as only five shared mutations were identified at contact sites with the ACE2 receptor, and all were found in 10 or fewer sequences. Of these, one mutation, at position 489, was synonymous and found in three sequences (0.02%). The others were nonsynonymous: G476S (*n* = 10 sequences, 0.05%), Y453F (*n* = 5, 0.02%), G446V (*n* = 3, 0.02%), and A475V (*n* = 2, 0.01%). To predict the potential immune pressure linked to T cell responses, we developed a T cell immunogenicity index which takes into account the CD8 and CD4 epitope repertoires in the structural proteins of SARS-CoV-2 (S, N, M, E) and the frequency of human leukocyte antigen (HLA) alleles or haplotypes in a given population. We found that sites with mutations, including 614 in S, were not colocalized with T cell epitopes frequently identified in different populations (*SI Appendix*, Figs. S6 and S7), and there was no significant relationship between the number of mutations and the immunogenicity index (*SI Appendix*, Fig. S8).

### Most Sites in the SARS-CoV-2 Genome Were under Purifying Selection.

**Fig. 3.**
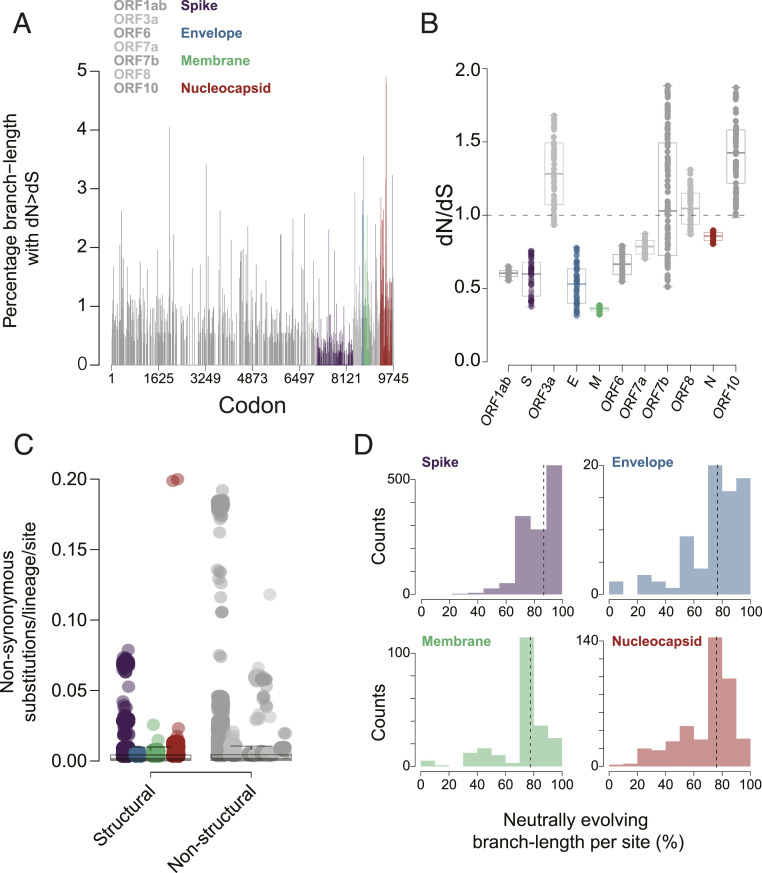
Evolution across the SARS-CoV-2 genome. (*A*) Bar plot of the average percentage of branch length under diversifying selection (dN/dS > 1) for each site. (*B*) Bar plot of dN/dS per gene (dN = dS is shown as dashed line). Error bars indicate SD across subsampled alignments. (*C*) Box plot of nonsynonymous substitutions per lineage per site across structural and nonstructural genes. Values across subsampled alignments for each gene are plotted. (*D*) Average percentage (over subsampled alignments) of branch lengths evolving under neutral (or negative) selection per site for each structural gene. Median values are shown by dashed lines.

Using phylogenetically informed models (as described above), we identified two sites, residue 614 in S and 13 in N, that were under diversifying selection in a majority of subsampled alignments. For each protein, subsampled alignments tended to have more sites under purifying selection (median = 7.34 ± 4.06% [±SD]) than under diversifying selection (3.10 ± 1.92%) (Mann−Whitney *U* test, *P* = 0.057; *SI Appendix*, Fig. S4) (purifying selection is indicative of a decrease in genetic diversity in the population). Likewise, for each codon separately, the proportion of each phylogeny (i.e., the percentage of total branch length) with dN/dS > 1 was small, indicating diversifying selection was episodic and limited (Fig. 3*A*). Global measures of dN/dS varied across genes, ranging from 0.35 ± 0.02 (M) to 1.43 ± 0.24 (ORF10), and were significantly lower for structural genes compared to nonstructural genes (Mann−Whitney *U* test, *P* = 0.042) (Fig. 3*B*). Per-lineage nonsynonymous substitution rates were comparable (Student’s *t* test, *P* = 0.218) in structural (0.0011 ± 0.021) and nonstructural (0.0012 ± 0.028) genes, although some subsampled alignments showed rates that could be a hundred times higher than the median over all alignments (Fig. 3*C*). Across structural proteins, mutations were disproportionately neutral: >70.3% of branch length evolved under neutral (or negative) selection for all sites, and over half of all branch length evolved under neutral (or negative) selection for >82.8% of sites (Fig. 3*D*) (29).

### No Evidence of Differentiation of the Viral Population.

**Fig. 4.**
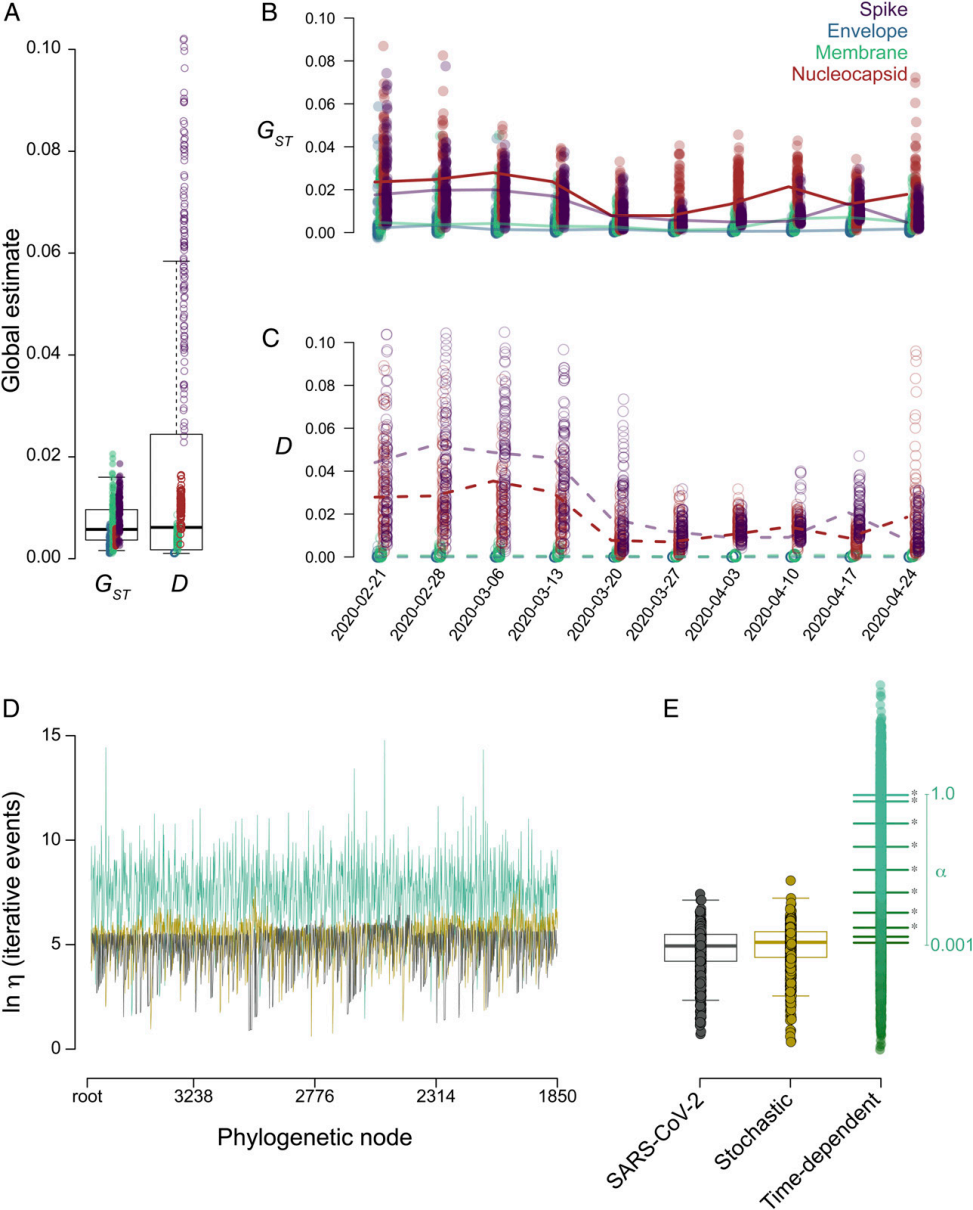
Limited evidence of adaptation of the viral population. (*A*–*C*) Bootstrapped global estimates of Nei’s G_ST_ and Jost’s D for population differentiation for each structural gene. (*A*) Estimates of Nei’s G_ST_ (closed circles) and Jost’s D (open circles) comparing sequences sampled from the Hubei province to sequences subsequently sampled globally. Estimates of (*B*) Nei’s G_ST_ and (*C*) Jost’s D comparing sequences sampled before or after a specific date. Lines connect the median estimates across datasets for each gene. (*D*) Ln-transformed phylogenetic η, indicative of the number of iterative events in the sampled subtree, for subtrees from each internal node (after the root) of a down-sampled SARS-CoV-2 whole-genome phylogeny (dark gray), of a phylogeny simulated under neutral parameters (gold), and of a phylogeny simulated under positive time-dependent rates (b(t) = 0.01e^0.4t^, green). (*E*) Box plot of ln-transformed phylogenetic η estimates across all down-sampled SARS-CoV-2 whole-genome phylogenies, phylogenies simulated under neutral parameters, and phylogenies simulated under different positive time dependencies, α. Asterisks indicate significant differences in mean values (Student’s *t* test, *P* < 0.05) between the SARS-CoV-2 and positive time-dependent phylogenies at each α.

While there was only limited evidence of diversification at selected sites, we also assessed whether subpopulations among the globally circulating viral population had become genetically differentiated over time. To do so, we used two measures of population differentiation, the G_ST_ and D statistics, which characterize changes in allele frequency across populations and can show fitness differences between subpopulations (30–32). Genetic distances between two subpopulations can range between 0 and 1, indicating no and complete differentiation, respectively. We initially compared 30 genomes sampled from the initial outbreak in Wuhan, China, with subsampled alignments of the 18,484 genomes sampled subsequently across the globe. Although distances varied across genes, the median genetic distance between these subpopulations was small for both G_ST_ (0.0049 ± 0.0047) and D (0.0053 ± 0.0272), indicating little differentiation between the initial outbreak and its global derivatives in the pandemic (Fig. 4*A*). We then compared subpopulations sampled before and after each consecutive week. Similarly, genetic distances between subpopulations were small for both G_ST_ (0.0058 ± 0.0096) and D (0.0098 ± 0.0650) and tended to narrow over time rather than diverge (Fig. 4 *B* and *C*). Signatures of host adaptation can also be seen in the branching patterns of viral phylogenies. Bursts in transmissibility are emblematic of increases in relative viral fitness and are reflected in imbalances in the phylogeny, which can be estimated at each internal node (*SI Appendix*, Figs. S9 and S10) (33–35). We estimated phylogenetic η (36, 37) at each internal node of the SARS-CoV-2 phylogeny reconstructed from subsampled (10%) alignments and compared the distribution of estimates through time to phylogenies simulated under models of neutral and positive time-dependent rates (b(t) = be^α(t)^). Simulation analyses demonstrated that this metric was robust against sampling fraction (*SI Appendix*, Fig. S10). The distribution of η in the SARS-CoV-2 phylogenies adhered to expectations of the neutral model and deviated significantly (Student’s *t* test, *P* < 0.001) from those of positive time-dependent rates for selection coefficients α ≥ 0.2 (Fig. 4 *D* and *E*). Together, the SARS-CoV-2 population and phylogenetic dynamics showed little evidence that the global spread of SARS-CoV-2 was related to viral fitness effects.

### Sequence Identity with Potential Vaccine Candidates.

**Fig. 5.**
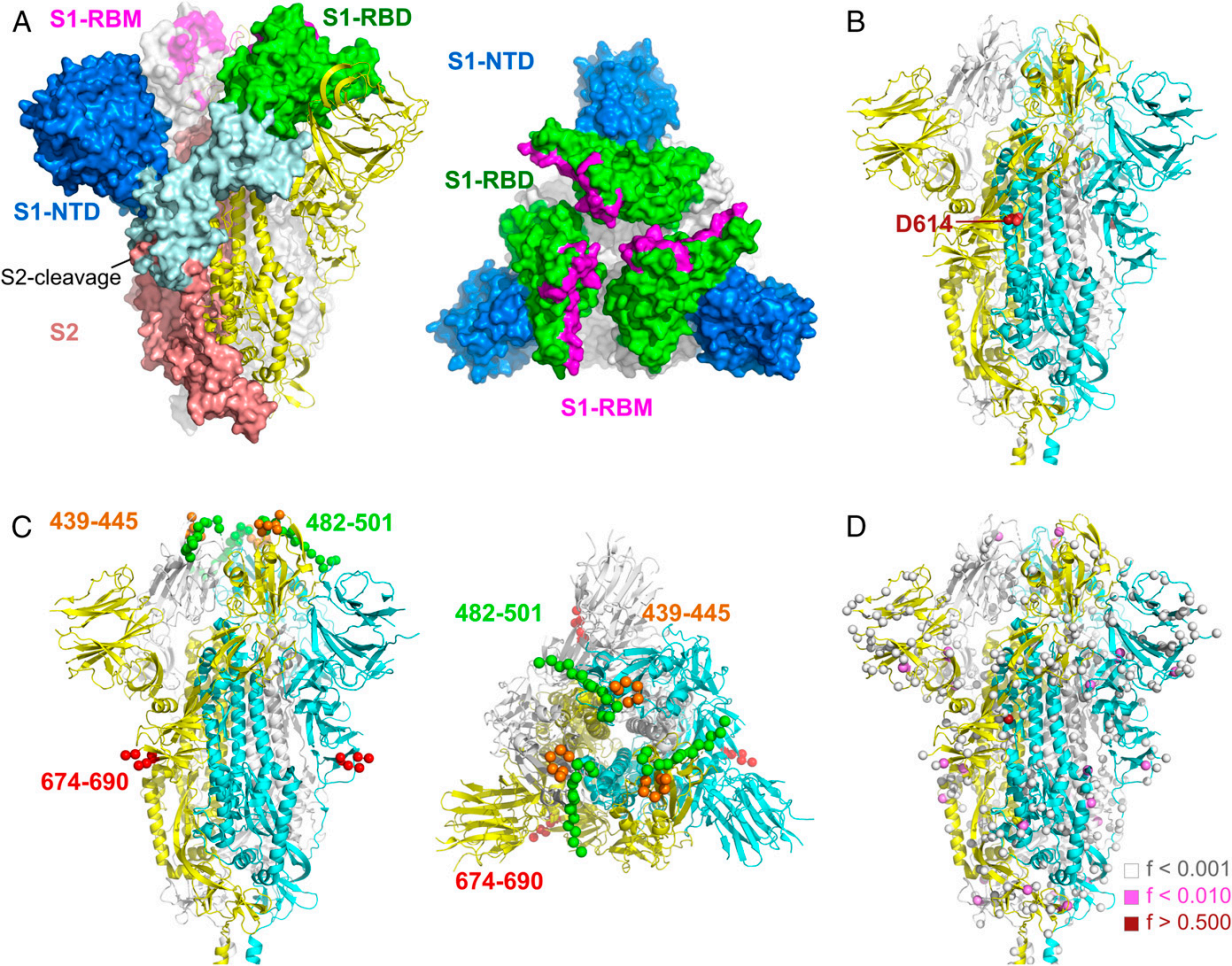
Mutations across SARS-CoV-2 S sequences. (*A*) Structure of SARS-CoV (5 × 58) (shown instead of SARS-CoV-2 for completeness of the Receptor Binding Motif [RBM]). (*B*–*D*) The three protomers in the closed SARS-CoV-2 S glycoprotein (Protein Data Bank ID code 6VXX) are colored in yellow, cyan, and white. Sites with mutations are shown as spheres. (*B*) Near-identity of potential vaccine candidates. The MRCA and Wuhan-Hu-1 reference sequences were identical, while the consensus derived from all circulating sequences showed a mutation (D614G). Site 614 is located at the interface between two subunits. (*C*) Sequence segments that differed between human and pangolin or bat hosts. Amino acid segments 439 to 455 and 482 to 501 impact receptor binding, while the 574 to 690 segment corresponds to the S2 cleavage site. (*D*) Sites with shared mutations across SARS-CoV-2 circulating sequences. The colors of the spheres correspond to the proportion of SARS-CoV-2 sequences that differed from the Wuhan-Hu-1 sequence (GISAID: EPI_ISL_402125, GenBank: NC_045512). Mutations that were found only in one or two sequences were not represented.

Typical vaccine design strategies rely on either 1) selecting sequences sampled from infected individuals or 2) computationally deriving sequences that cover the diversity seen across circulating sequences and are, in theory, optimal compared to an individual isolate (38). Computationally derived sequences include consensus and ancestral sequences, such as the most recent common ancestor (MRCA) of a set of sequences. We inferred the MRCA corresponding to 1) SARS-CoV-2 S sequences sampled from Wuhan within the first month of the epidemic, 2) all currently circulating SARS-CoV-2 sequences, and 3) all SARS-CoV-2 sequences together with closely related sequences sampled from pangolins (*n* = 6) and a bat. There were 17 mutations between the human MRCA and the human−bat MRCA and 44 mutations between the human MRCA and human−pangolin MRCA. Overall, three segments in S reflected significant variability across species (AA 439 to 445, 482 to 501, and 676 to 690) (Fig. 5 *A* and *C* and *SI Appendix*, Fig. S11) (39). In contrast, when considering only human sequences, SARS-CoV-2 diversity was limited: Both MRCAs (derived from early sequences from Wuhan or from all circulating sequences) were identical to the initial reference sequence Wuhan-Hu-1. Comparing these sequences to the consensus sequence derived from all of the sequences sampled to date, there was only one mutation: D614G (Fig. 5*B*). Fig. 5*D* illustrates that mutations found across circulating S sequences were rare: Besides D614G (found in 69.4% of sequences), the next most frequent substitution is found in 1.96% of sequences (synonymous), with sequences sampled from infected individuals, on average, 0.55 mutations away from the consensus sequence (consisting of 0.12 synonymous and 0.43 nonsynonymous mutations). Across the genome, there were, on average, 4.05 nucleotide mutations per individual genome when compared to the consensus, with only P4715L and D614G found in >50% of sequences.

## Discussion

There remains an urgent need for a SARS-CoV-2 vaccine as a primary countermeasure to mitigate and eventually contain the spread of COVID-19. The virus’s S glycoprotein makes an attractive vaccine target because it plays a key role in mediating virus entry and is known to be immunogenic (40). Neutralizing antibody responses against S have been identified in SARS-CoV-2−infected individuals (2), and several clinical trials for a SARS-CoV-2 vaccine will test S as an immunogen. While we focused on S, our comparative analyses of other proteins yielded similar conclusions: A randomly selected SARS-CoV-2 sequence could be used as a vaccine candidate, given the similarity of any sequence to the computationally derived optimum vaccine candidate (as defined by the MRCAs or consensus sequence based on all circulating sequences). Vaccines developed using any of these sequences should, theoretically, be effective against all circulating viruses. Vaccine developers could consider designing a vaccine insert with the D614G mutation in S, as this mutation has become dominant worldwide. While mutations that become fixed are often linked to the host immune pressure, this seems unlikely for the SARS-CoV-2 mutation D614G. Because this residue lies at the interface between two subunits, it would not be expected to be part of a critical epitope for vaccine-mediated protection (Fig. 4). As such, pseudoviruses with D614G were as susceptible to neutralization as those with the initial residue D614 (25). A mutation, S612L, that emerged in MERS-CoV after passaging the virus in the presence of two antibodies (in 5/15 clones after 20 passages) (41) warrants the evaluation of the analogous D614G mutation in SARS-CoV-2 for its ability to interfere with the recognition of a distal epitope. A more direct path to viral escape from antibody recognition would be mutations in the RBD, as described for influenza (42, 43). Importantly, we found no mutation in the RBD that was present in more than 1% of SARS-CoV-2 sequences (highest frequency was 0.2% N439K); such rare variants are unlikely to interfere with vaccine efficacy.

In the context of rare SARS-CoV-2 mutations, the rapid spread of the D614G mutation is singular and has led authors to hypothesize that viruses with D614G may have enhanced fitness (24). The strongest evidence of a biological effect for this mutation comes from recent reports of an increase in in vitro infectivity or cell entry for pseudoviruses with D614G (25–28). Additional work is needed to evaluate whether the increase in infectivity in vitro translates to increased transmissibility (spread) of SARS-CoV-2 across humans, as there is not necessarily a linear relationship between the two. For example, SARS-CoV mediates cell entry more efficiently than SARS-CoV-2 (with or without the D614G S mutation) (26). Hence, it would be important to understand whether, controlling for epidemiological factors, there are higher reproduction numbers associated with viruses carrying the D614G mutation. While a preliminary comparison of the lineages with either D or G in Washington State did not indicate an obvious advantage for D614G mutants, as they found similar maximal values for the effective reproduction number (https://github.com/blab/ncov-wa-phylodynamics), additional comparisons in different geographic locations should be informative.

Correlating in vitro findings with clinical phenotypes can be complicated. During the Ebola outbreak of 2013–2016, some fixed mutations were suspected to confer an advantage to the virus. Specifically, an A82V mutation in the glycoprotein, which, like S for SARS-CoV-2, is critical for the virus entry into host cells, was associated with an increase in infectivity (44–46). Yet, effects varied across cell types (47), and no phenotypic differences were associated with the mutations when viruses were evaluated in vivo in mouse and nonhuman primate models (48), highlighting the difficulty in linking biological mechanisms to outcomes at the population level. So far, no causal association has been identified between the presence of D614G and disease severity (24).

These findings, together with our results, illustrate that mutations can spread through the population without necessarily having a selective advantage, especially at the beginning of an epidemic when most individuals are susceptible. Mutations occur more frequently after a host switch, and even slightly deleterious mutations may have an opportunity to spread. Hence, the main signal in our study was one of purifying selection that can ultimately eliminate mildly deleterious mutations. Our analyses showed limited evidence of diversifying selection, with comparable substitution rates in structural proteins versus nonstructural proteins (under a selection paradigm, structural proteins which are essential for viral entry and the target of the host immune response would have higher rates than the nonessential proteins), low estimates of genetic differentiation following the initial outbreak, and phylogenetic patterns adhering to a neutral process of evolution.

These data indicate that epidemiologic factors could be sufficient to explain the global spread of mutations such as D614G. A founder effect means that these mutations were likely exported to SARS-CoV-2 naive areas early in the outbreak and therefore given the opportunity to spread widely. As such, on January 28, 2020, a virus carrying the D614G mutation, which was rare among sequences from China, was identified in Germany. Host and environmental factors permitted the establishment of a sustained cluster of infections that propagated this mutation until it became dominant among European sequences and then globally (Fig. 2). We found no evidence that the frequent identification of this mutation was caused by convergent selection events that would have occurred in multiple individuals. Further analyses are needed to characterize the biologic mechanisms behind the spread of the D614G mutation.

In summary, our results indicate that, so far, SARS-CoV-2 has evolved through a nondeterministic, noisy process and that random genetic drift has played a dominant role in disseminating unique mutations throughout the world. Yet, it is important to note that founder effects do not exclude that the D614G can confer distinguishing properties in terms of protein stability, infectivity, or transmissibility. SARS-CoV-2 was only recently identified in the human population—a short time frame relative to adaptive processes that can take years to occur. Although we cannot predict whether adaptive selection will be seen in SARS-CoV-2 in the future, the key finding is that SARS-CoV-2 viruses that are currently circulating constitute a homogeneous viral population. Viral diversity has challenged vaccine development efforts for other viruses such as HIV-1, influenza, or Dengue, but these viruses each constitute a more diverse population than SARS-CoV-2 viruses (*SI Appendix*, Fig. S12). We can therefore be cautiously optimistic that viral diversity should not be an obstacle for the development of a broadly protective SARS-CoV-2 vaccine, and that vaccines in current development should elicit responses that are reactive against currently circulating variants of SARS-CoV-2.

## Materials and Methods

### Sequence Data.

Sequences were downloaded from GISAID (https://www.gisaid.org/). A full list, along with the originating and submitting laboratories (GISAID_acknowledgment_table_20200518.xls), is available at https://www.hivresearch.org/publication-supplements.

### Sequence Processing and Filtering.

All SARS-CoV-2 sequences available on GISAID as of May 18, 2020 (*n* = 27,989) were downloaded and deduplicated where possible, and those missing accurate dates (that is, only recording the month and/or year) were removed. Sequences were processed using the Biostrings package (version 2.48.0) in R (49). Sequences known to be linked through direct transmission were removed, and only the sample with the earliest date (chosen at random when multiple samples were taken on the same day) was retained. Sequences were then aligned with Mafft v7.467 using the -addfragments option to align to the reference sequence (Wuhan-Hu1, GISAID accession EPI_ISL_402125) (50). Insertions relative to Wuhan-Hu-1 were removed, and the 5′ and 3′ ends of sequences (where coverage was low) were excised, resulting in an alignment consisting of the 10 ORFs. Any sequences with less than 95% coverage of the ORFs (i.e., >5% gaps) were removed, and 30 homoplasic sites likely due to sequencing artifacts identified by de Maio et al. were masked (https://github.com/W-L/ProblematicSites_SARS-CoV2/blob/master/archived_vcf/problematic_sites_sarsCov2.2020-05-27.vcf).

To identify individual sequences that were much more divergent than expected, given their sampling date, which likely reflected sequencing artifacts rather than evolution, we obtained a tree using FastTree v2.10.1 compiled with double precision under the general time reversible (GTR) model with gamma heterogeneity (51). This tree was rooted at the reference sequence, and root-to-tip regression was performed following TempEst using the ape package in R (52, 53). Outliers were defined as sequences that had studentized residuals greater than 3, and were removed.

Sequences from the United Kingdom corresponded to nearly half of the sequences (*n* = 12,157/25,671, 47%) of this filtered dataset. To avoid overrepresentation of the UK sequences and bias in subsequent analyses, we investigated the effect of downsampling sequences on the mean Hamming distance and identified the minimum number of sequences required to recover the mean corresponding to the full distribution (*SI Appendix*, Fig. S1). A subsample of 5,000 sequences satisfied these criteria, and also ensured that there were fewer sequences from the United Kingdom than from the United States (*n* = 5,398), reflecting the epidemiology. These 5,000 sequences were sampled randomly, with weight proportional to the number of UK sequences collected on that day.

After these filtering steps, the alignment used for subsequent analyses included 18,514 sequences.

### Global Phylogeny and Evolution.

The global phylogeny was reconstructed in FastTree v2.10.1 compiled with double precision under the GTR model with gamma heterogeneity (51), and rooted at the reference sequence. The tree was visualized using ggtree in R (54). Lineages were defined using PANGOLIN (Phylogenetic Assignment of Named Global Outbreak LINeages), with lineages with >200 taxa as of the May 19 summary being highlighted in the tree (22) (https://github.com/cov-lineages/lineages). The number of polymorphic sites was calculated as the number of sites which had at least one mutation relative to the reference sequence, Wuhan-Hu-1, ignoring gaps and ambiguities.

### Pairwise Distance Comparisons.

For each pair of sequences, we calculated the Hamming distance as the number of sites that are different after removing sites with ambiguities and/or gaps. For computational efficiency, given the size of the alignment, this was implemented in parallel in C++, using Bazel (https://bazel.build/) to build on a Linux system. This implementation is available to download at https://www.hivresearch.org/publication-supplements.

### Subsampling Gene Alignments.

Alignments for each gene were subsampled for sequence and phylogenetic analyses. Each gene alignment was randomly subsampled 100 times per collection date at 5%, 10%, 20%, 30%, and 40%. When fewer than 10 sequences were available for a collection date, all sequences were taken. Median Hamming distances were computed for each set of subsampled alignments. These were bootstrapped 100,000 times, and 95% CIs were estimated and compared to the median Hamming distance for the fully sampled alignment.

### Global and Site-Specific Nonsynonymous and Synonymous Substitution Rates.

Alignments subsampled at 10% 100 times were used to estimate substitution rates. For the set of subsampled alignments for each gene, a mixed-effect likelihood method was used to estimate nonsynonymous (dN) and synonymous (dS) substitution rates globally and at each codon (29). Maximum-likelihood phylogenies were constructed for each alignment using the software IQ-TREE (55) under a best-fit model determined with ModelFinder (56) to prime the dN and dS estimates before branch length optimization. This step serves to expedite the optimization process. Branch length optimization was done with a MG94 model [which is the only model available for this analysis (29)]. The proportion of each phylogeny evolving under neutral (or negative) selection was determined from the mixture density across lineages for each site, assuming different dN and dS along each branch (57). On the same set of subsampled alignments and phylogenies, a fixed-effects likelihood method was used on internal branches to identify sites under pervasive diversifying selection and to estimate global dN/dS (58). Known biases associated with calculating dN/dS on exponentially growing populations (59) were counterbalanced by subsampling phylogenies, as the typical approach to address this bias, which is to ignore terminal branches, would considerably diminish the power of the analysis to detect any significant result. As *P* values from the fixed-effect likelihood method are uncorrected, results were not averaged over *P* values; rather, given that *P* value calculations are conservative for this analysis (58), sites were considered to be under pervasive diversifying selection if their *P* value was <0.1 in ≥50% of alignments, which would account for a typical 5% false discovery rate (58).

### Global and Gene-Specific Population Differentiation.

Alignments subsampled at 10% 100 times were used to estimate population differentiation. The genetic differentiation of subpopulations within sampled sequences was calculated on each gene separately using Nei’s (30) G_ST_. Because comparisons between subpopulations of different sizes can bias genetic differentiation estimates (60), genetic differentiation was also calculated using Jost’s (31) D, which accounts for differences in genetic heterogeneity between subpopulations and is intended to correct for biases in the size of the subpopulations. Both statistics were computed with the mmod package (32) in R (v3.6.1). For each gene, statistics were calculated over 100 bootstrapped samples for each subsampled alignment. Subpopulations were defined in two ways. First, sequences originating from the initial outbreak in the Hubei province (30 sequences) were compared to all other sequences within a subsampled alignment. Second, a 1-wk sliding window was designed to compare all sequences sampled prior to a collection date (subpopulation 1) to all sequences sampled after the same collection date (subpopulation 2). The first collection date for subpopulation 1 was February 14, 2020, the week after the last sequence from the Hubei province was sampled (February 8, 2020), The window was designed to terminate when <30 sequences were available in subpopulation 2.

### Time-Dependent Estimates of Phylogenetic Diversification.

Time-dependent estimates of phylogenetic diversification were measured by extracting the branches descending from each internal node (above the root) of each phylogeny and calculating the peak height (η) of the spectral density profile of the graph Laplacian of each subtree, which is a measure of the density of branching events (36, 37). The code to perform the analysis is available for download at https://www.hivresearch.org/publication-supplements.

#### Simulation analyses.

Phylogenies were simulated using a time-forward branching process under constant birth rates (b(t)=b) and time-dependent birth rates ($b\left(t\right)=be^{\alpha t}$) for *b* = 0.01, 0.03, 0.05, 0.07, and 0.09 and *α* = ±0.01, ±0.11, ±0.21, ±0.31, and ±0.41, for 20, 220, 420, 620, and 820 tips, and for 1, 11, 21, 31, and 41 time units. Simulated phylogenies were downsampled at 0%, 10%, 30%, 50%, and 70%. For each scenario, 100 phylogenies were simulated. Time-dependent diversification (i.e., η across subtrees) was calculated for each phylogeny simulated under each scenario. Simulations were conducted using the R packages RPANDA (R Phylogenetic ANalyses of DiversificAtion) (61) and ape (53).

#### Comparisons to SARS-CoV-2 phylogeny.

Phylogenies downsampled at 10% from the full (18,514 tips) SARS-CoV-2 genome phylogeny (following the subsampling strategy described above) were used to calculate the phylogenetic η for each subtree (above the root) for each downsampled phylogeny. Neutral phylogenies were simulated under stochastic branching by randomly sampling from the distribution of branch lengths from one downsampled SARS-CoV-2 phylogeny. This was iterated across all downsampled SARS-CoV-2 phylogenies. Positive time-dependent phylogenies were simulated using a time-dependent process ($b\left(t\right)=0.01e^{\alpha t}$) for *α* = 0.001, 0.1, 0.2, 0.3, 0.4, 0.5, 0.6, 0.7, 0.8, and 1, with branch lengths restricted to the distribution of branch lengths from one downsampled SARS-CoV-2 phylogeny. This was iterated across all downsampled SARS-CoV-2 phylogenies for each α. Neutral and positive time-dependent phylogenies were simulated with a 10% sampling fraction. Polytomies were randomly resolved. Simulations were conducted using the R packages RPANDA (61) and ape (53).

### Ancestral S Protein Sequence Reconstruction.

Ancestral S protein sequences were reconstructed from an amino acid alignment of 30 SARS-CoV-2 sequences sampled from the Hubei province, a coronavirus sampled from bat (Yunnan RaTG13), and six SARS-CoV-2-like coronaviruses sampled from pangolins using maximum posterior probability and returning a unique residue at each site assuming a Jones-Taylor-Thornton (JTT) model with gamma heterogeneity (62). The JTT model was the most appropriate model available in the software (62). The bat sequence was retrieved from GenBank, and the pangolin sequences were retrieved from GISAID (63). A sliding window of 10 amino acids (and a step of 1 amino acid) was used to compare the cumulative number of mutations in the human−bat and human−bat−pangolin ancestors with respect to the human ancestral sequence. Median values for each window were compared to a null window (computed as a normal distribution of 10 values with a mean equal to the mean value across the entire S protein, 0.046 mutations) using a one-tailed *t* test. An alignment including the reconstructed sequences is available at https://www.hivresearch.org/publication-supplements.

### Prediction of CD4+ and CD8+ T Cell Epitopes.

CD4+ and CD8+ T cell epitopes were predicted for four SARS-CoV-2 structural proteins: S (accession YP_009724390), N (accession YP_009724397), M (accession YP_009724393), and E (accession YP_009724392). CD4+ T cell epitopes were predicted using a server that predicts binding of peptides to any MHC molecule of known sequence using artificial neural networks, NetMHCIIPan 4.0 (64) with a peptide length of 15. MHC class II HLA alleles of HLA-DQB1, plus the haplotypes of HLA-DPA1-DPB1 and HLA-DQA1-DPB, were selected for predictions if they had frequencies of >1/1,000 in known allele/haplotype distributions (http://17ihiw.org/17th-ihiw-ngs-hla-data/). If multiple peptides had the same core, the peptide with the strongest binding score was selected for analysis. CD8+ T cell epitopes were predicted using NetMHCPan 4.1 (64) with a peptide length of 9. MHC class I HLA alleles of HLA-A, HLA-B, and HLA-C were selected if they were classified as common (frequency ≥ 1/10,000) in any of the populations in the database CIWD 3.0 (Common, Intermediate and Well-Documented HLA Alleles in World Populations) (65). Epitopes predicted as strong binders (with predicted binding affinities below 50 nM) were selected for analyses.

### T Cell Immunogenicity Index.

For each site in a predicted epitope, the immunogenicity index was defined as the sum of the frequency of the HLA alleles or haplotypes restricting the corresponding epitope (multiple epitopes can be predicted at a given site in a protein). Total frequencies from CIWD 3.0 were used as the frequencies of the corresponding MHC class I HLA alleles (HLA-A, HLA-B, and HLA-C), and the global frequencies from http://17ihiw.org/17th-ihiw-ngs-hla-data/ were used as the frequencies of the corresponding MHC class II HLA alleles or haplotypes (HLA-DQB1, HLA-DPA1-DPB1, and HLA-DQA1-DPB). This procedure was repeated using the frequencies of MHC alleles or haplotypes in different subpopulations listed in the above HLA frequency dataset.

### Statistical Analyses.

For comparisons of mean values in normally distributed data, Student’s *t* test was used. When data were not normal, the Mann−Whitney *U* test was used. Shapiro−Wilk tests were used to determine normality. Differences in data distributions were estimated using the Kolmogorov−Smirnov test.

## Supplementary Material

Supplementary File

## Data Availability

Data and code are available at https://www.hivresearch.org/publication-supplements. All study data are included in the article and *SI Appendix*.
