# Supplementary material for: A SARS-CoV-2 vaccine candidate would likely match all currently circulating variants
Source: Proc Natl Acad Sci U S A. 2020 Aug 31;117(38):23652–62. doi: 10.1073/pnas.2008281117 (PMC7519301; doi:10.1073/pnas.2008281117)
Supplement: Supplementary File [file pnas.2008281117.sapp.pdf]

PNAS
www.pnas.org

Supplementary Information for

A SARS-CoV-2 vaccine candidate would likely match all currently circulating variants

Bethany Dearlove[1,2,3,4]†, Eric Lewitus[1,2,3,4]†, Hongjun Bai[1,2,3,4], Yifan Li[1,2,3,4], Daniel B. Reeves[5], M. Gordon Joyce[1,3], Paul T. Scott[1], Mihret F. Amare[1,3], Sandhya Vasan[2,3,4], Nelson L. Michael[4], Kayvon Modjarrad[1,4]†*, Morgane Rolland[1,2,3,4]†*,

*Corresponding authors: mrolland@hivresearch.org (M.R.) and kayvon.modjarrad.civ@mail.mil (K.M.)

This PDF file includes:

Figures S1 to S12

Other supplementary materials for this manuscript include the following:

Datasets and code are available at: https://www.hivresearch.org/publication-supplements

www.pnas.org/cgi/doi/10.1073/pnas.2008281117

Supplementary Information

Table of contents

Fig. S1. Sequence curation 3

Fig. S2. Narrow distribution of Hamming distances across SARS-CoV-2 genome 4

Fig. S3. Hamming distances across subsampled alignments at different sampling fractions 5

Fig. S4. Sites under diversifying and purifying selection 6

Fig. S5. Location of the Spike D614G mutation 7

Fig. S6. CD8+ T cell immunogenicity index and mutations identified across circulating sequences for SARS-CoV-2 structural proteins 8

Fig. S7. CD4+ T cell immunogenicity index and mutations identified across circulating sequences for SARS-CoV-2 structural proteins 9

Fig. S8. Lack of relationship between the number of mutations in SARS-CoV-2 structural proteins and the T cell immunogenicity index 10

Fig. S9. Schematic description of the estimation of viral fitness through time 11

Fig. S10. Simulated estimates of viral fitness through time 12

Fig. S11. Ancestral reconstruction of the SARS-CoV-2 spike sequence 13

Fig. S12. Comparison of the diversity found across circulating SARS-CoV-2 or HIV-1 sequences 14

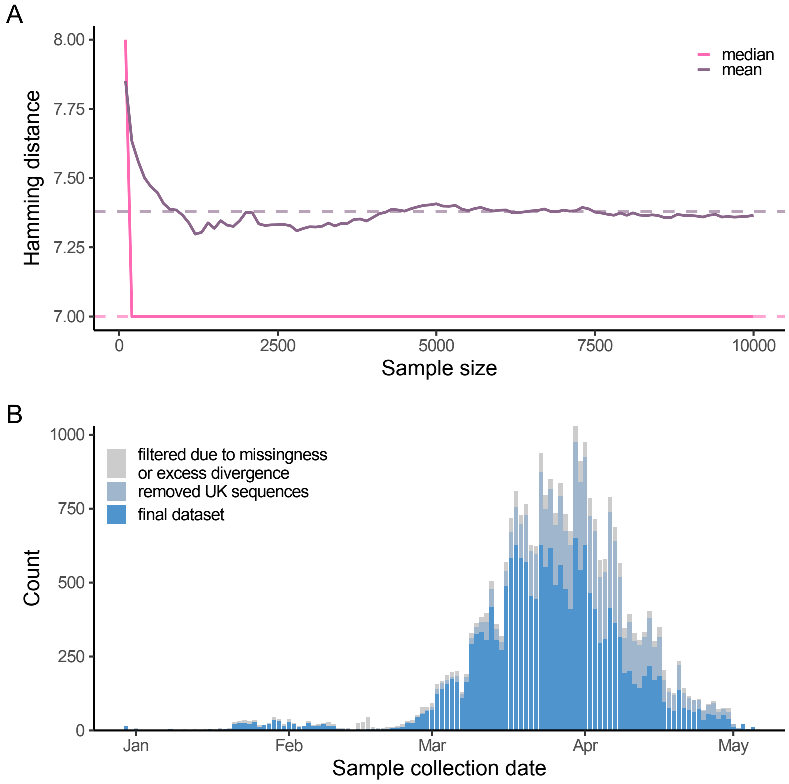

Fig. S1. Sequence curation. A dataset of 18,514 SARS-CoV-2 independent sequences was used for analyses. 27,989 sequences were downloaded from GISAID on May 18th, 2020. Sequences from the UK corresponding to a large fraction of the dataset (n=12,157/25,671, 47%) were downsampled. (A) A sample of 5,000 UK sequences was considered representative based on the analyses of subsets of sequences sampled randomly. (B) Distribution of sequences in the final dataset (n = 18,514 sequences) compared to the original dataset of 27,989 sequences (387 sequences are not represented for lack of precise collection date).

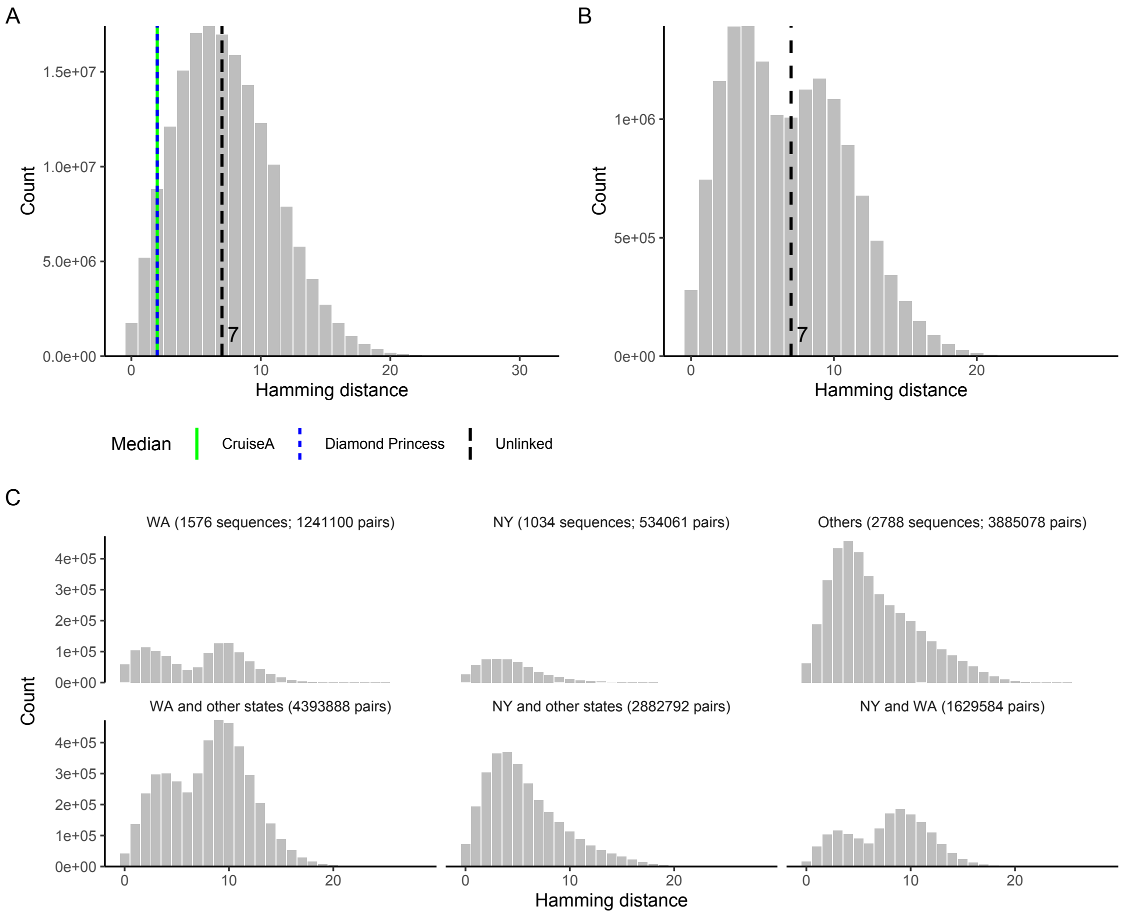

Fig. S2. Narrow distribution of Hamming distances across SARS-CoV-2 genomes. The x-axis represents the number of nucleotide substitutions between two genomes and the median is shown as a dashed line. (A) Hamming distances were calculated across 18,608 genome sequences. The median is reported for all unlinked individuals (n = 18,514, median = 7) as well as for linked cases from Cruise A (n = 25, median = 2) or from the Diamond Princess cruise (n = 69, median = 2). (B) Hamming distances across 5,398 genome sequences sampled in the US. (C) Hamming distances calculated for subsets of sequences corresponding to Washington (WA) and New York (NY) States.

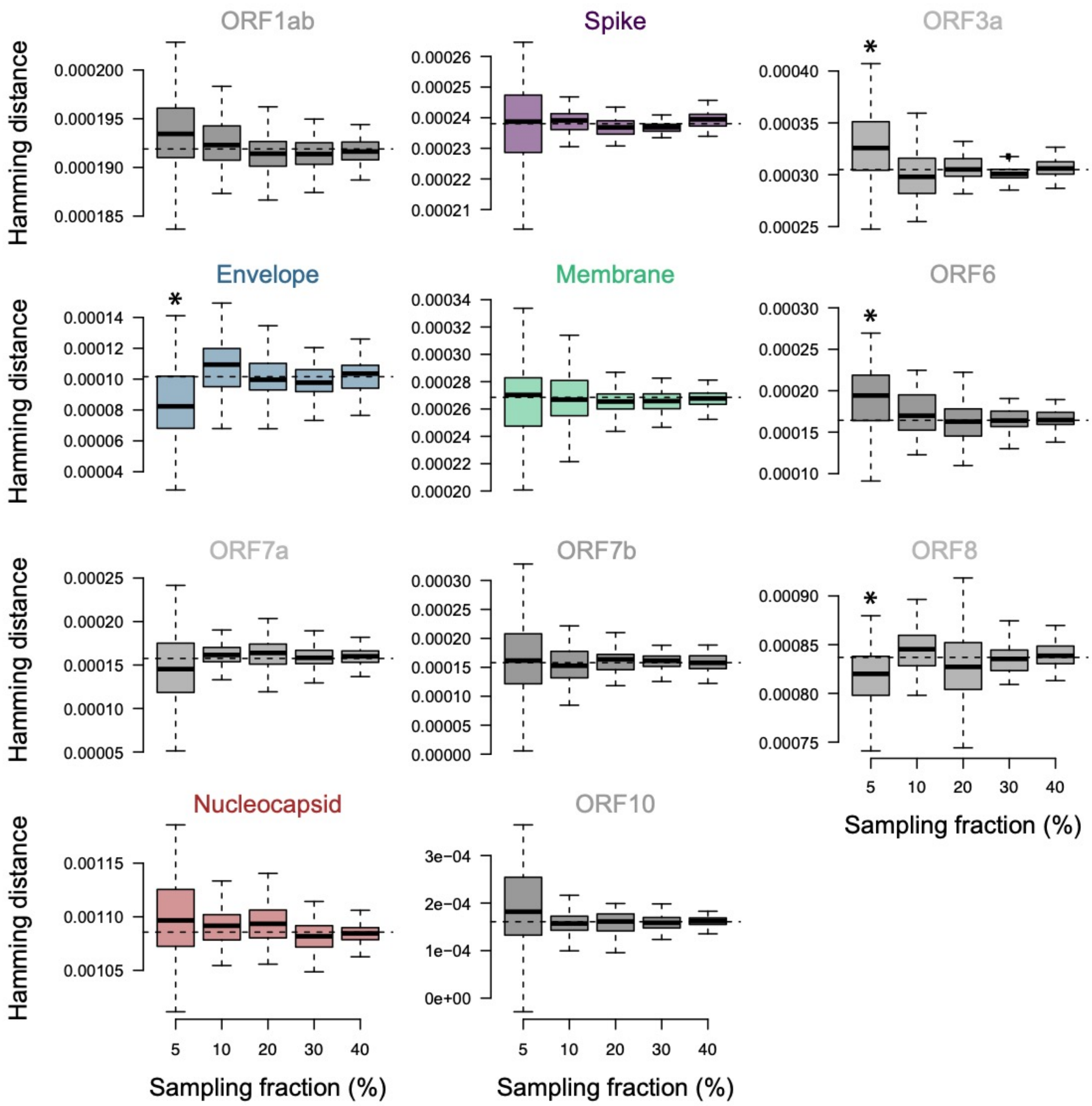

Fig. S3. Hamming distances across subsampled alignments at different sampling fractions. Boxplots are shown as median estimates (as a proportion of sequence length) across subsampled alignments per gene. Dashed lines indicate the median hamming distance for the complete alignment for each gene. Asterisks denote statistically significant differences between the distribution of distances in the sampling fraction and that of the complete alignment (Mann-Whitney U test, P<0.05).

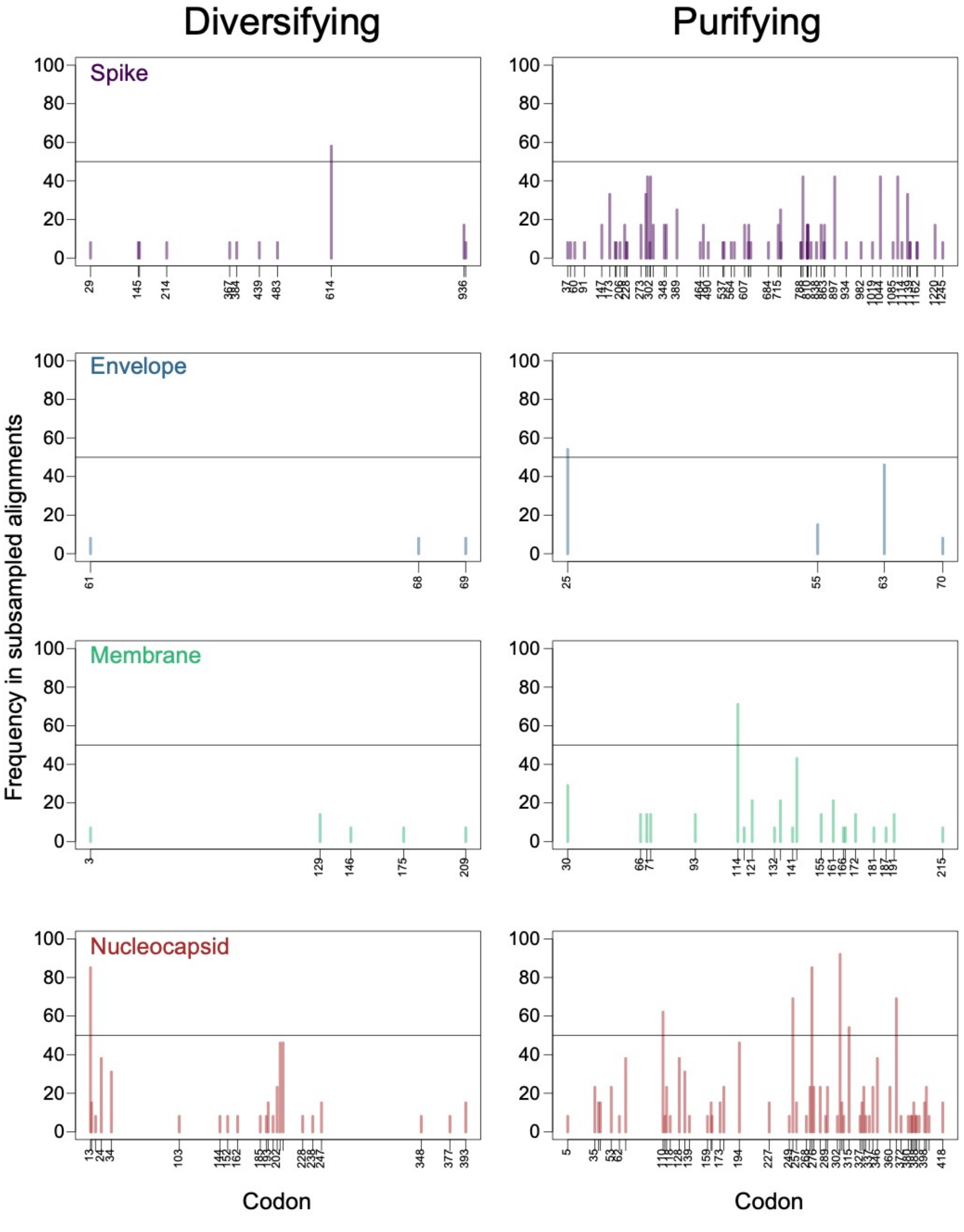

Fig. S4. Sites under diversifying and purifying selection. The frequency of codons under pervasive diversifying (left) and purifying (right) selection across subsampled alignments per gene. The dashed line shows the threshold for 50% frequency.

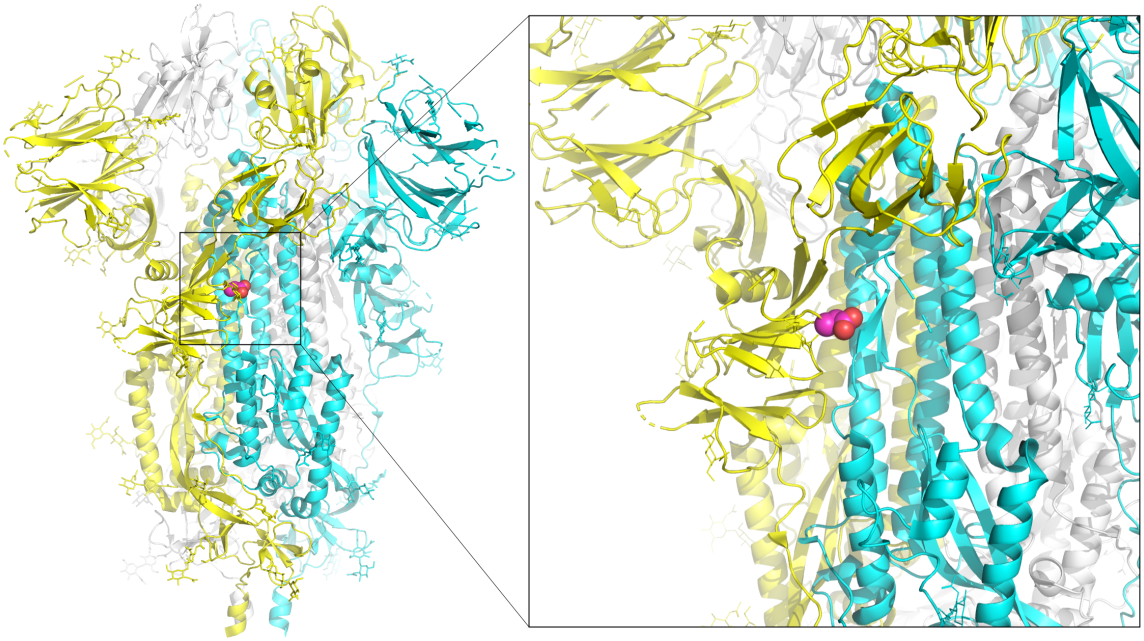

Fig. S5. Location of the Spike D614G mutation. The mutation D614G is at the interface between two subunits.

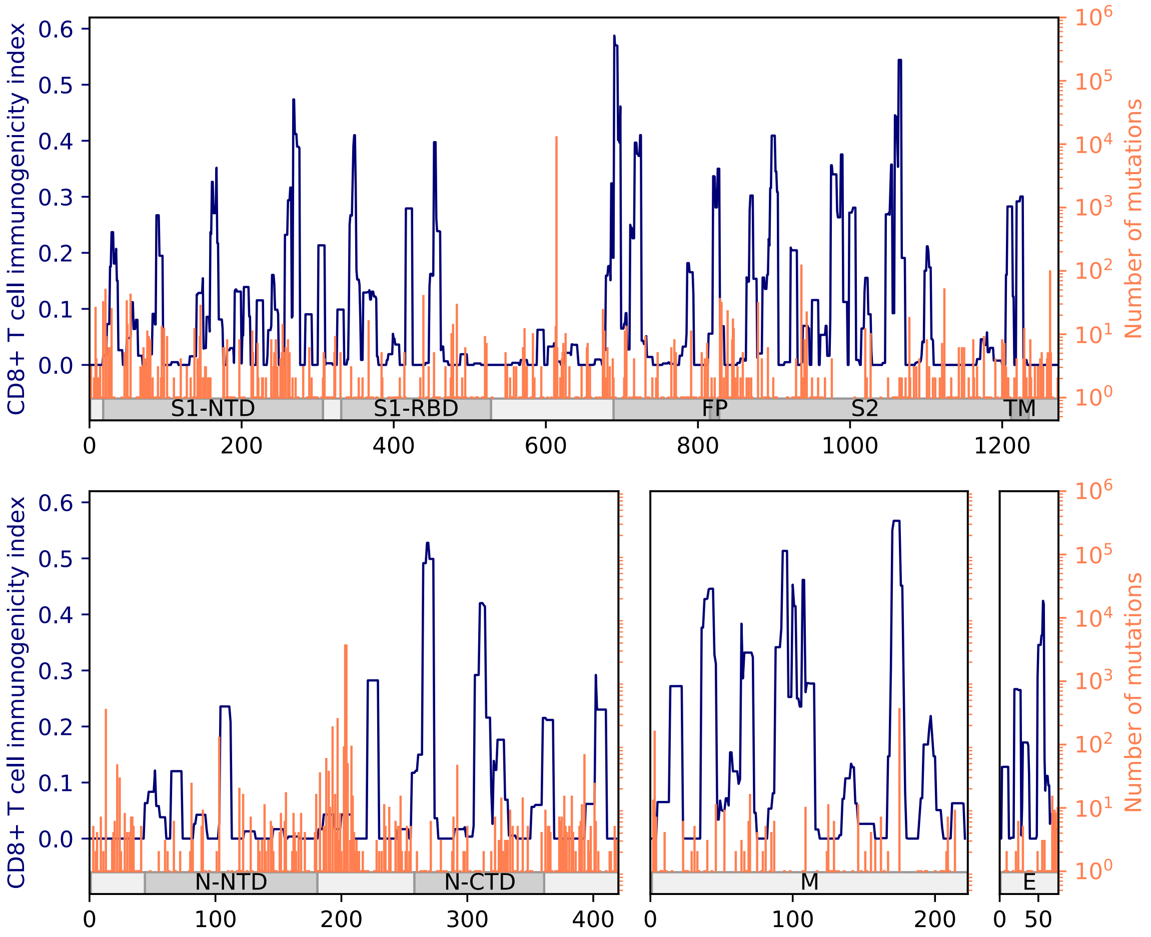

Fig. S6. CD8+ T cell immunogenicity index and mutations identified across circulating sequences for SARS-CoV-2 structural proteins. The number of amino acid mutations was calculated using sequences isolated from Wuhan as a reference. Only mutations found in more than one circulating sequence were considered.

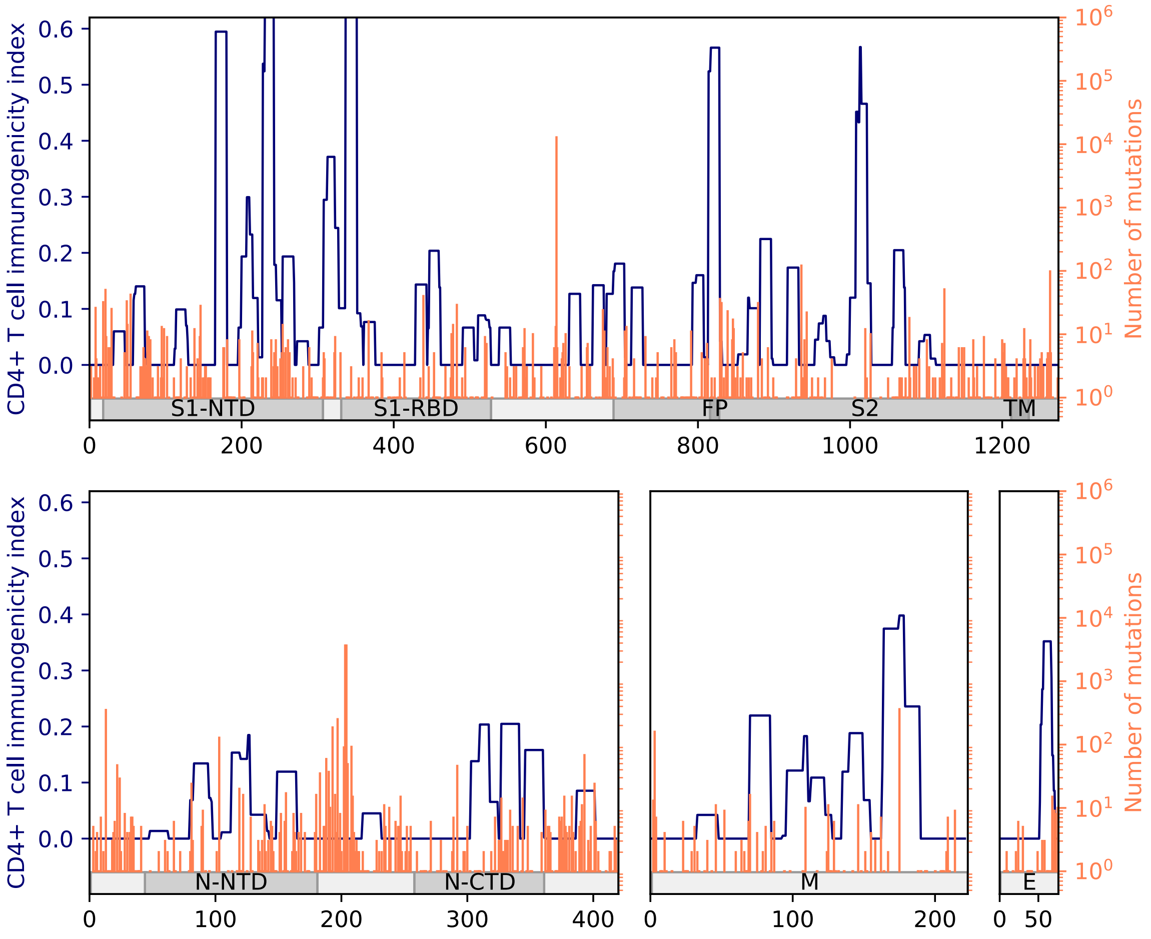

Fig. S7. CD4+ T cell immunogenicity index and mutations identified across circulating sequences for SARS-CoV-2 structural proteins. The number of amino acid mutations was calculated using sequences isolated from Wuhan as a reference. Only mutations found in more than one circulating sequence were considered.

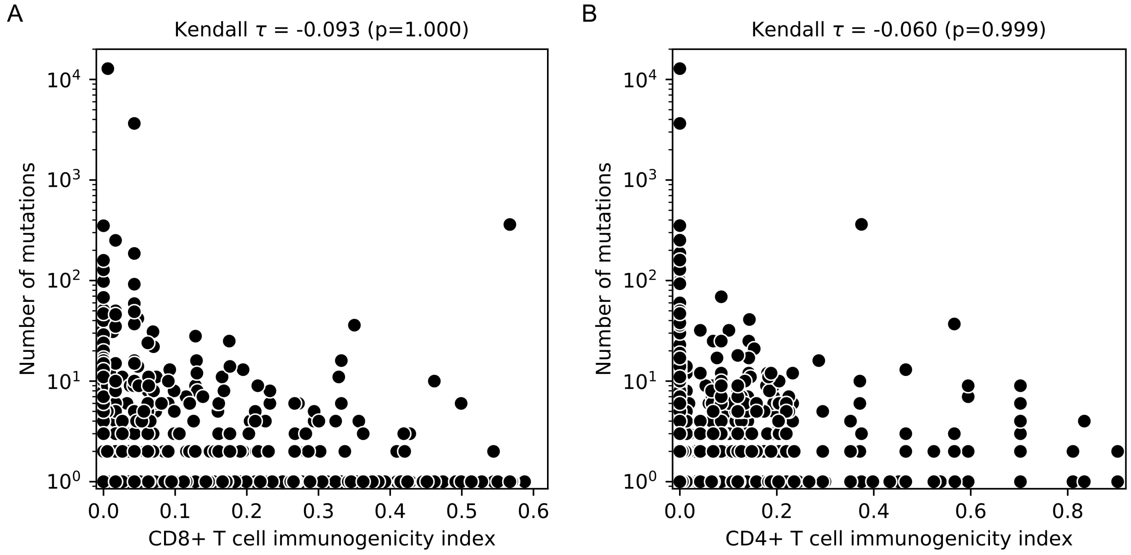

Fig. S8. Lack of relationship between the number of mutations in SARS-CoV-2 structural proteins and the T cell immunogenicity index. Number of mutations as a function of the CD8+ (A) and CD4+ (B) T cell immunogenicity index. To show the y-axis as a logarithmic scale, we added 1 to the number of mutations at each site (sites with no change = 1).

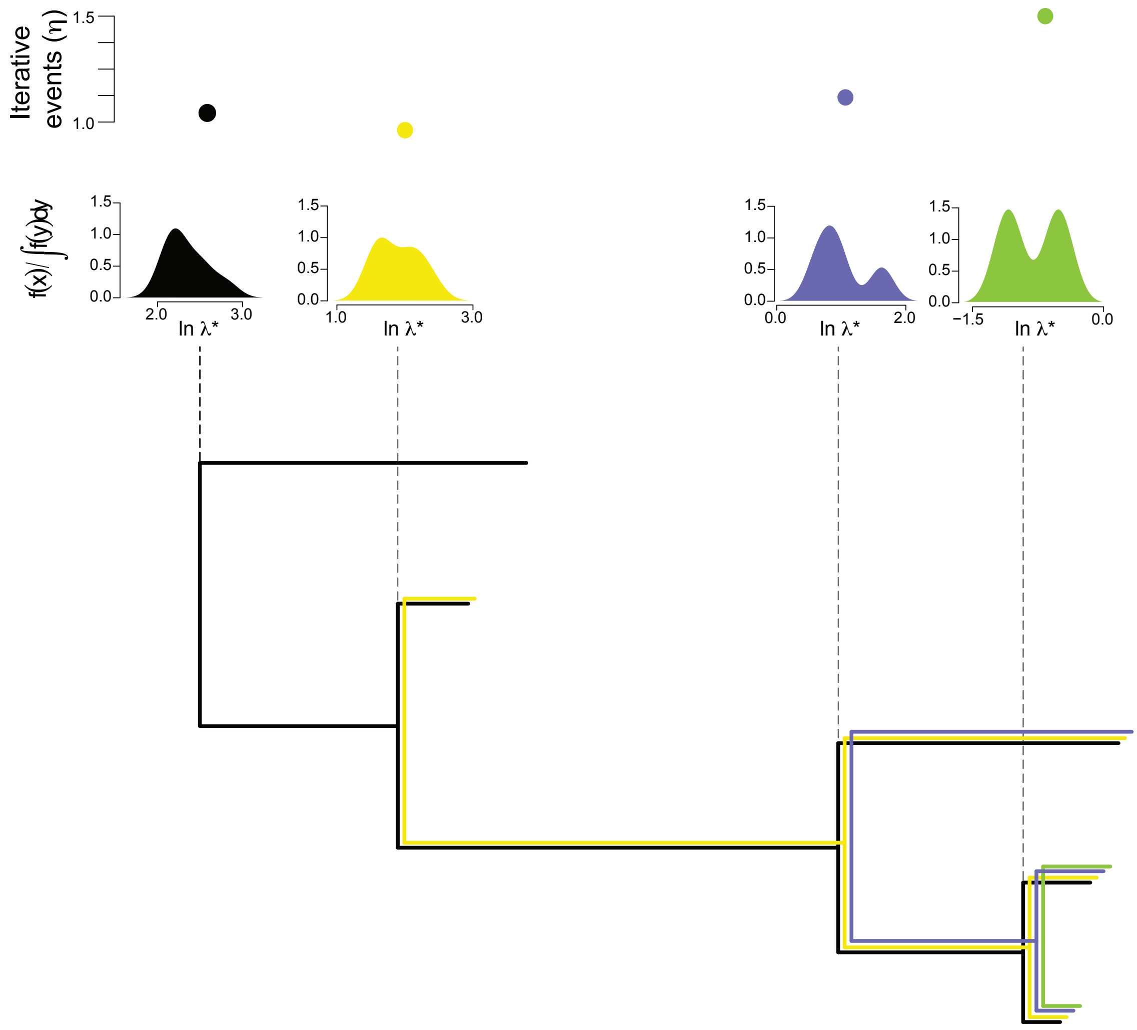

Fig. S9. Schematic description of the estimation of viral fitness through time. Cartoon example of estimating η, indicative of iterative branching events in the phylogeny over time. (top panel) η estimates and (middle panel) spectral density profiles of the graph Laplacian constructed from subtrees descending from a given node to the tips (within a phylogeny) are shown.

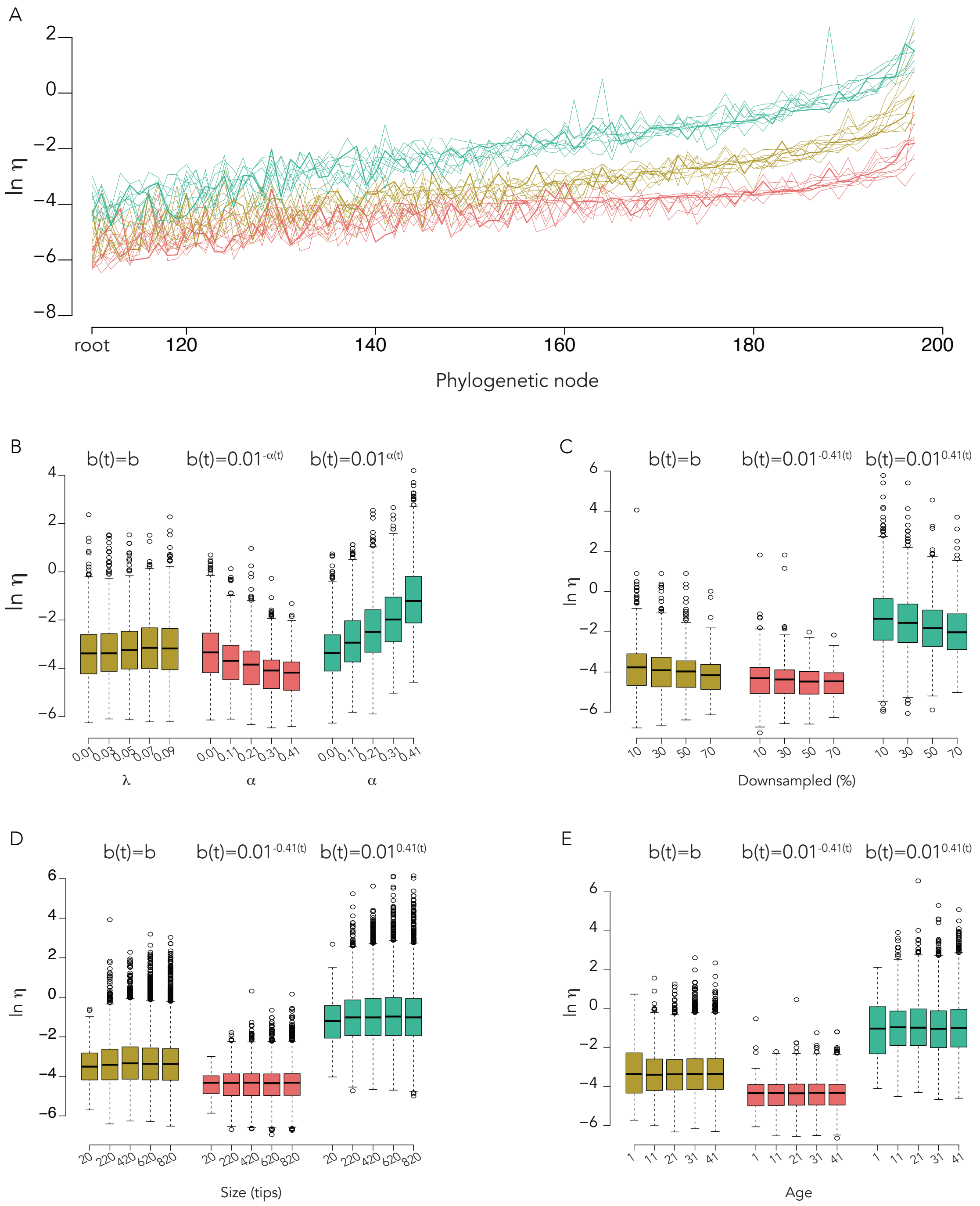

Fig. S10. Simulated estimates of viral fitness through time. (A) Ln-transformed η estimates for phylogenies simulated under constant rates (gold), negative time-dependent rates (tomato), and positive time-dependent rates (teal). Estimates are shown for 50 simulations under each model with 100% phylogenetic sampling. (B-E) Boxplots of ln-transformed η estimates for constant-rate, negative time-dependent, and positive time-dependent phylogenies with varying (B) birth-rates, b, and dependencies, α, (C) constant birth-rates and dependencies, but varying sampling fractions, (D) constant birth-rates and dependencies, but varying number of simulated tips, and (E) constant birth-rates and dependencies, but varying simulated ages. Simulated models are indicated above each boxplot.

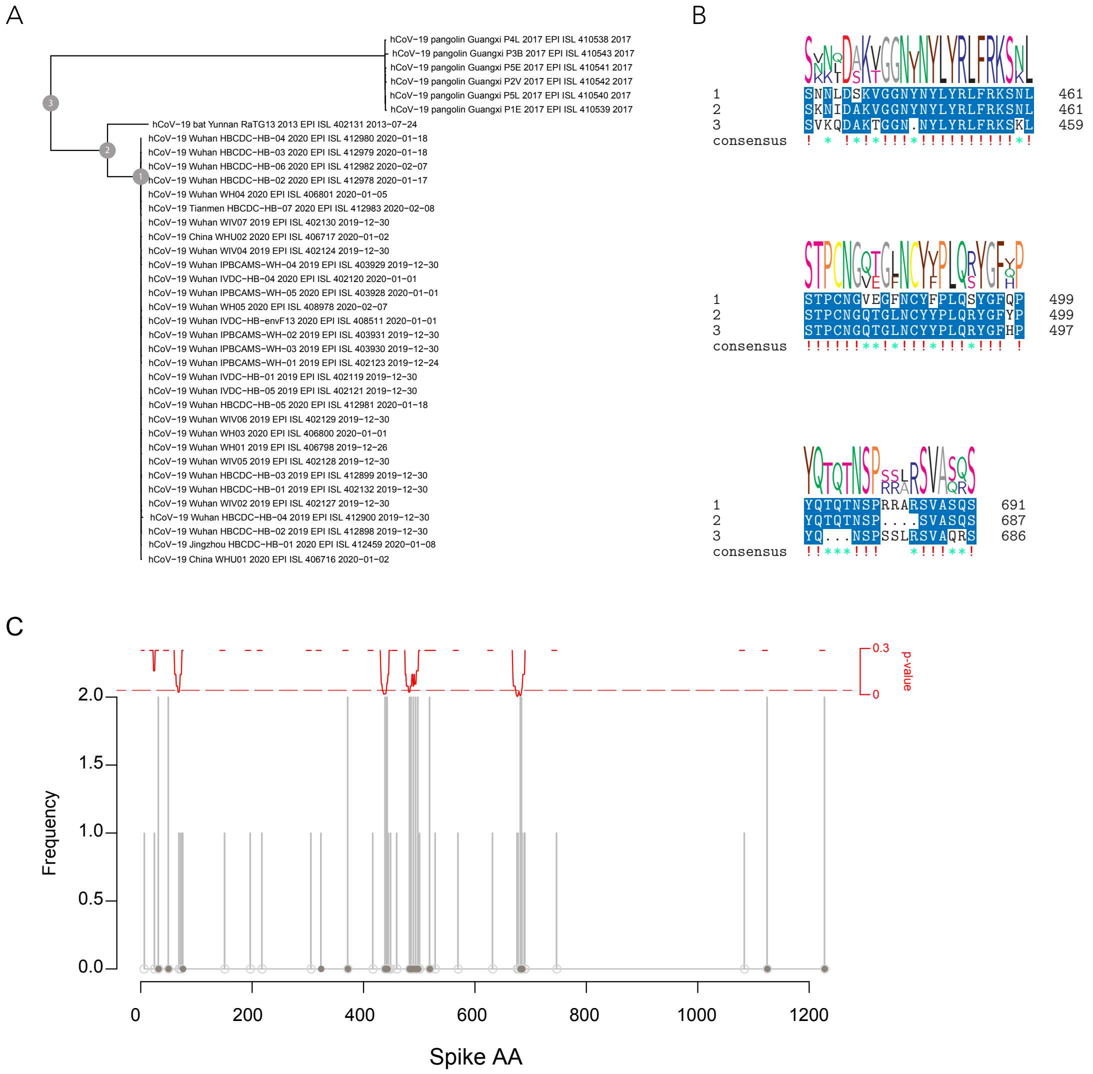

Fig. S11. Ancestral reconstruction of the SARS-CoV-2 spike sequence. (A) Phylogenetic tree reconstructed from SARS-CoV-2 genomes corresponding to sequences sampled from Hubei province, six sequences sampled from Pangolins between 2017-2019, and one sequence from a bat collected in 2013. Human (1), human and bat (2), and human, bat, and pangolin (3) ancestral nodes are indicated. (B) Amino acid sequence alignments from the most diverse regions between the three reconstructed ancestral sequences. (C) Frequency of changes from the consensus across the sequences in (B). P-values are shown for distribution densities that deviate significantly (based on a one-tailed t-test) from a null distribution with a mean value equal to the mean across all Spike protein sites.

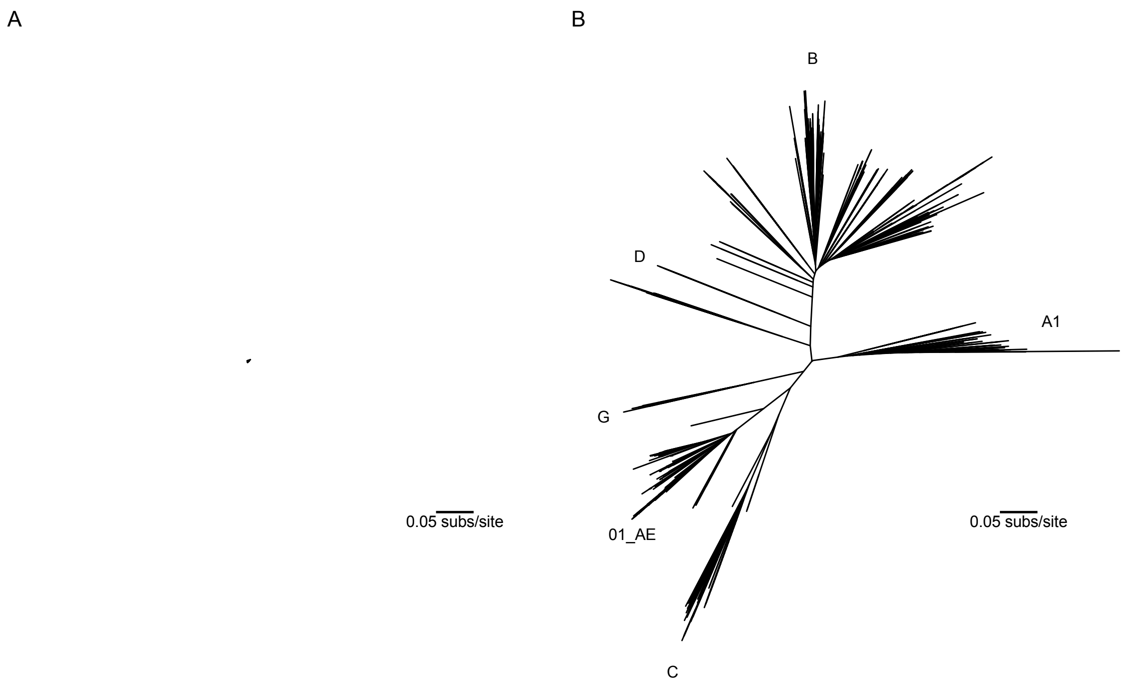

Fig. S12. Comparison of the diversity found across circulating SARS-CoV-2 or HIV-1 sequences. The trees show the diversity found across SARS-CoV-2 spike sequences (n = 18,514) (A) and across HIV-1 envelope sequences sampled in 2010 (n = 1,067) (B). HIV-1 sequences were downloaded from the LANL HIV-1 database (www.hiv.lanl.gov). The purpose of this figure with both trees shown on the same scale is to illustrate the extent of diversity that needs to be covered by an HIV-1 vaccine compared to what a SARS-CoV-2 vaccine will need to cover.
